# Causal Relationship Between Depression and Traumatic Brain Injury: A Two‐Sample Mendelian Randomization Analysis

**DOI:** 10.1002/brb3.70669

**Published:** 2025-07-07

**Authors:** Shiping Wang, Lei Pan, Binyang Wang, Qianwen Ruan, Ying Shi, Tong Sun, Xu Yang, Lei Zhang, Xiaohua Ke, Geng Li, Meihua Qiu, Chuanxiong Li

**Affiliations:** ^1^ Department of Rehabilitation Medicine The Affiliated Hospital of Yunnan University Kunming Yunnan Province China; ^2^ Second Affiliated Hospital of Yunnan University of Chinese Medicine Kunming Yunnan Province China; ^3^ Yunnan University of Chinese Medicine Kunming Yunnan Province China; ^4^ Kunming Medical University Kunming Yunnan China; ^5^ The Second Affiliated Hospital of Kunming Medical University Kunming Yunnan Province China; ^6^ Affiliated Sichuan Provincial Rehabilitation Hospital of Chengdu University of TCM Chengdu Sichuan Province China; ^7^ Department of Rehabilitation Medicine, Shanghai Fourth People's Hospital, School of Medicine Tongji University Shanghai China

**Keywords:** bidirectional MR, causal inference, depression, genetic instrumental variables, psychiatric comorbidity, TBI

## Abstract

**Background:**

Traumatic brain injury (TBI) and depression are major global health burdens, yet their bidirectional causal relationship remains unclear.

**Objective:**

To explore the causal relationship between depression and TBI, and to clarify whether depression is one of the potential risk factors for TBI and whether TBI is one of the pathogenic factors for depression.

**Methods:**

This bidirectional two‐sample Mendelian randomization (MR) analysis investigated causal relationships between depression (*n* = 170,756) and TBI (*n* = 3193) using genome‐wide association study (GWAS) summary statistics. Genetic instruments were selected as single nucleotide polymorphisms (SNPs) significantly associated with exposures (depression/TBI) and outcomes (TBI/depression) at genome‐wide significance (*P* < 5 × 10⁻⁶). The inverse variance weighted (IVW) method under fixed‐effects and multiplicative random‐effects models served as the primary analytical approach, with Cochran's* Q *test evaluating SNP heterogeneity. To address horizontal pleiotropy, MR‐Egger regression and MR‐PRESSO(MR Pleiotropy RESidual Sum and Outlier)outlier correction were applied. Sensitivity analyses included weighted median, penalized weighted median, maximum likelihood estimation, and leave‐one‐out validation to ensure robustness. All analyses were conducted using the TwoSampleMR package in R (v4.3.2), with effect estimates reported as odds ratios (OR) and 95% confidence intervals (CI).

**Results:**

MR analyses revealed bidirectional causal relationships between depression and TBI. In forward analyses, depression increased TBI risk across multiple IVW frameworks (fixed‐effects IVW: OR = 1.137, 95% CI = 1.019–1.271, *P* = 0.022; multiplicative random‐effects IVW: OR = 1.137, 95% CI = 1.014–1.277, *P* = 0.028), corroborated by maximum likelihood estimation (OR = 1.137, 95% CI = 1.017–1.274, *P* = 0.024). Reverse analyses demonstrated TBI's causal effect on depression through IVW models (fixed‐effects: OR = 1.083, 95% CI = 1.036–1.131, *P* < 0.001; multiplicative random‐effects: OR = 1.083, 95% CI = 1.043–1.124,*P* < 0.001) and penalized weighted median methods (OR = 1.079, 95% CI = 1.018–1.145, *P* = 0.011). Robustness was confirmed by null heterogeneity (Cochran's Q: forward *P* = 0.209, reverse *P* = 0.596) and absence of horizontal pleiotropy (MR‐PRESSO: forward *P* = 0.218, reverse *P* = 0.672; MR‐Egger intercepts: forward *P* = 0.661, reverse *P* = 0.874). All effect estimates remained stable in sensitivity analyses, supporting unconfounded causal inference.

**Conclusion:**

Our MR analyses robustly demonstrate bidirectional causality: depression is a risk factor for TBI (OR = 1.137, 95% CI = 1.019–1.271), and TBI subsequently increases depression risk (OR = 1.083, 95% CI = 1.036–1.131), advocating integrated clinical monitoring.

## Graphical abstract

Causal Link: Depression → TBI via MR Analysis

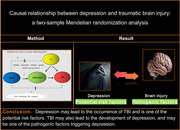

**Abbreviations**
CI: confidence intervals
GWAS: genome‐wide association study
IVW: inverse variance weighted
MAOIs: Monoamine Oxidase Inhibitors
MR: Mendelian randomization
MR‐PRESSO: Mendelian Randomization Pleiotropy RESidual Sum and Outlier
OR: Odds ratios
Q: Cochran's Q Statistic
SNPs: Single nucleotide polymorphisms
SNRIs: Serotonin‐Norepinephrine Reuptake Inhibitors
SSRIs: Selective Serotonin Reuptake Inhibitors
TBI: Traumatic brain injury
TCAs: Tricyclic Antidepressants

## 1 Introduction

TBI is caused by external forces leading to brain damage, characterized by high mortality and disability rates (Gilmore and Karceski 2010). Preventing TBI is a critical public health priority, and understanding its risk factors is essential for developing targeted prevention strategies. Established risks include traffic accidents, falls, external violence, and self‐inflicted injuries (Dewan et al. 2018; Jiang et al. 2019; Barbiellini Amidei et al. 2024; Dams‐O'Connor et al. 2023). Emerging evidence suggests bidirectional associations between TBI and factors such as low educational attainment, nicotine dependence, social engagement, and aggression. For instance, pre‐injury aggression may predispose individuals to TBI, whereas post‐injury neurobehavioral changes can also increase aggression (Matei et al. 2022).

Depression, a common psychiatric disorder, is primarily characterized by persistent anhedonia and a depressed mood (Malhi and Mann 2018). Current research confirms its bidirectional associations with multiple diseases. Epidemiological studies have shown significant comorbidity with hypertension (Cai et al. 2022), insomnia (Riemann et al. 2020), diabetes mellitus (Basiri et al. 2023), cardiovascular diseases (El‐Battrawy et al. 2021), cerebrovascular diseases (Pan et al. 2011), and obesity (Milaneschi et al. 2019). Conversely, clinical evidence indicates that conditions such as stroke (Robinson and Jorge 2016), sleep disorders (Zhang et al. 2022), chronic pain (Ferrarelli 2017), and hypothyroidism (Bode et al. 2021) may increase an individual's risk of depressive episodes. Regarding the causal relationship between depression and TBI, existing observational studies have identified correlations (McGuire et al. 2018; Keatley et al. 2023). However, inherent limitations, such as unmeasured confounding factors and potential detection biases in observational designs, prevent definitive causal inferences.

To validate the research question, this study employs MR, an epidemiological method for causal inference. MR utilizes genetic variants as instrumental variables to proxy exposures and assess their causal effects on outcomes. Genetic variants, defined as heritable DNA sequence alterations affecting phenotypic traits, primarily include SNPs and chromosomal variations. SNPs represent nucleotide substitutions (e.g., T→C) at specific genomic positions, with alternative nucleotides termed alleles (e.g., T and C alleles at a SNP locus). During gametogenesis, homologous chromosomes segregate following Mendel's first law (segregation), while non‐homologous chromosomes assort independently per Mendel's second law (independent assortment). This genetic randomization mechanism, mirroring the random allocation in randomized controlled trials (RCTs), effectively minimizes confounding biases. By leveraging this principle, MR enables robust elucidation of causal relationships between exposures and clinical outcomes (Weith and Beyer 2023).

Through bidirectional two‐sample MR, this study rigorously evaluates the potential causal interplay between depression and TBI, circumventing biases plaguing conventional observational studies.

## 2 Materials and Methods

This study employed genetic variants strongly associated with depression and traumatic brain injury (TBI) from publicly available GWAS datasets as instrumental variables. The causal relationship between these conditions was primarily investigated through IVW regression. To validate the result robustness, we performed comprehensive sensitivity analyses including weighted mode, simple mode, maximum likelihood, penalized weighted median, Cochran's *Q* test, MR‐PRESSO, and MR‐Egger approaches. As all GWAS data were derived from previously approved studies with documented ethical clearance by originating institutions, no additional ethics review was required for this secondary analysis.

### 2.1 Study Design

**FIGURE 1:**
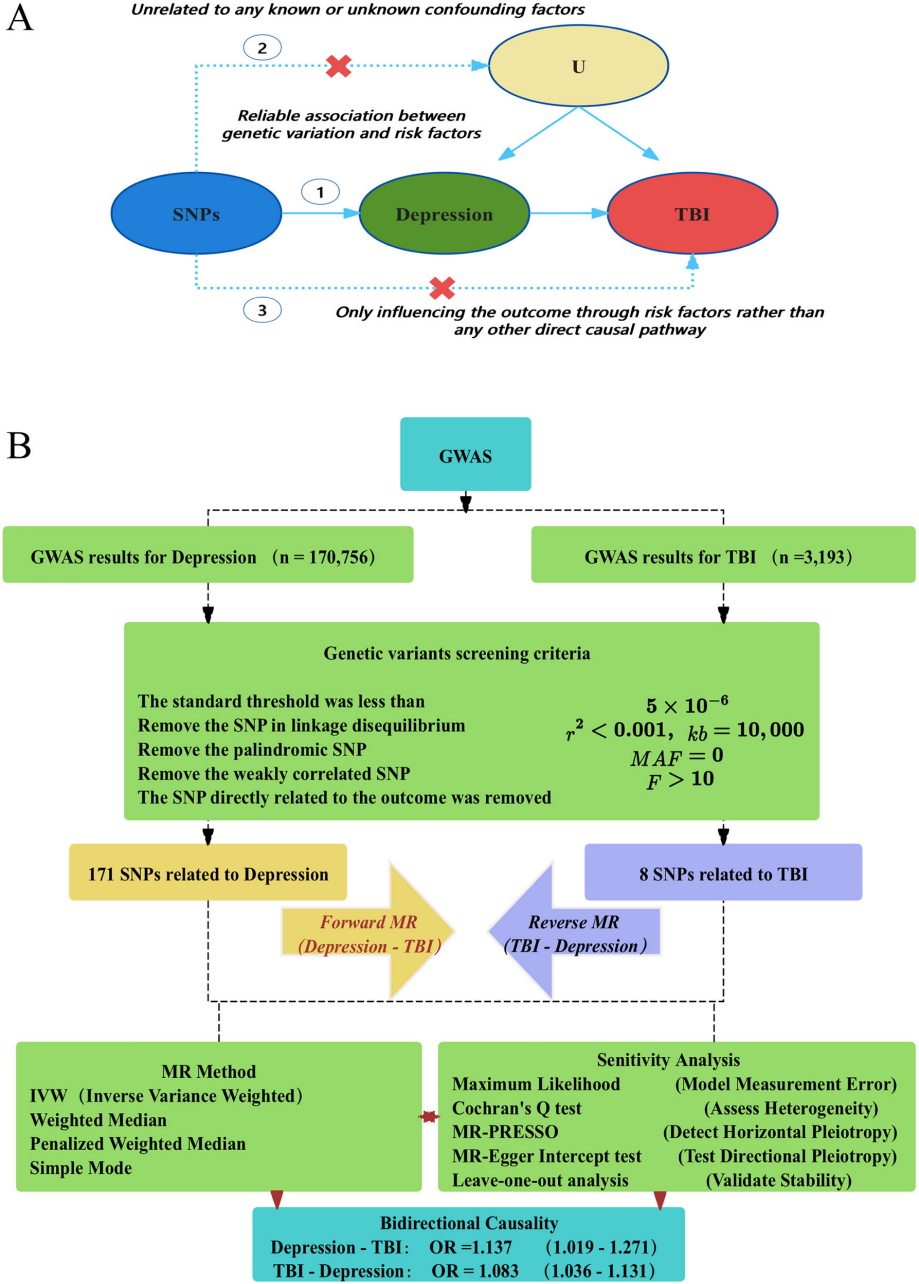
Schematic overview of the bidirectional two‐sample MR study design and assumptions. (A) three core MR assumptions: strong association between genetic variants and exposures (relevance assumption), independence of genetic variants from confounders (independence assumption), and exclusive influence of genetic variants on outcomes through exposures (exclusion restriction assumption); and (B) flowchart of forward MR (depression → TBI) and reverse MR (TBI → depression) analyses, illustrating GWAS data sources, SNP screening criteria (linkage disequilibrium removal, exclusion of weak instruments), and analytical methods. Final bidirectional causality results (depression → TBI: OR = 1.137; TBI → depression: OR = 1.083) were derived using the IVW approach. * This figure was created by the authors based on standard MR methodologies described in (Weith and Beyer 2023; Fang et al. 2024; Burgess et al. 2013).

MR design must satisfy three core assumptions (Figure 1A).

1. Relevance Assumption—The selected genetic variants as instrumental variables must exhibit a robust association with the exposure factors (e.g., depression or TBI).
2. Independence Assumption—These genetic variants should remain independent of both known and unknown confounding factors.
3. Exclusion Restriction Assumption—The genetic variants influence the outcome exclusively through the exposure factors, with no alternative causal pathways.

The bidirectional MR workflow, including SNP selection criteria and analytical methods, is summarized in Figure 1B.

The validity of genetic variants as instrumental variables may be influenced by biological mechanisms, genetic co‐inheritance, and population stratification. While definitive proof of these assumptions is unattainable, their plausibility can be evaluated through sensitivity analyses and empirical tests. For instance, assessing whether the association between genetic variants and the outcome diminishes after adjusting for exposure levels, performing individual causal effect estimations using multiple independent genetic variants, and leveraging biological evidence to substantiate the role of selected variants in the hypothesized pathway.

### 2.2 Data Sources

Using GWAS data as the primary source, the study focused on individuals of European ancestry, with both male and female cohorts included. Depression‐associated genetic instruments were derived from a large‐scale GWAS comprising 170,756 participants. After quality control (linkage disequilibrium clumping, r^2^ < 0.001), 171 genome‐wide significant SNPs were identified at a threshold of *P* < 5 × 10⁻⁶. For TBI, data from 3193 European‐ancestry cases yielded 8 SNPs meeting the same significance threshold (*P* < 5 × 10⁻⁶) and instrument strength criteria (F‐statistic > 10). Participant demographics (e.g., ancestry, sex distribution) remained consistent across forward and reverse MR analyses.

### 2.3 Selection and Validation of the SNPs

The selection of depression/TBI‐associated SNPs at a *P* < 5 × 10⁻⁶ (compared to the conventional *P* < 5 × 10⁻⁸) reflects an optimal balance between instrument availability and type I error control under limited sample size (*n* = 3193; projected IVs < 10 at stricter thresholds). This empirical thresholding strategy is anchored in:

(1) Prior MR studies showing that relaxed thresholds with sensitivity analyses maintain validity (Burgess et al. 2013);
(2) All SNPs had F‐statistics > 10, minimizing weak instrument bias;
(3) Consistency across pleiotropy‐robust methods (weighted median, MR‐Egger).

All SNPs exhibited F‐statistics > 10, confirming strong instrument validity. Sensitivity analyses (e.g., MR‐Egger intercept tests) further ensured that pleiotropic effects did not bias our estimates.

### 2.4 Statistical Analysis of the Two‐Sample MR

To rigorously assess the bidirectional causal relationships between depression and TBI, we employed a comprehensive suite of MR methods. These approaches were selected to address specific challenges in our dataset, such as heterogeneity, pleiotropy, and the limited sample size of the TBI cohort (*n* = 3193). Below, we detail the rationale and application of each method within the context of this study.

#### 2.4.1 Primary Analysis: IVW Method

The IVW method served as our primary analytical framework. This approach aggregates SNP‐specific Wald ratios (SNP‐outcome association divided by SNP‐exposure association) using inverse variance weights, assuming balanced pleiotropy (i.e., pleiotropic effects cancel out across SNPs) and no measurement error in SNP‐exposure associations. While IVW provides high statistical power, its validity depends on the absence of directional pleiotropy. To mitigate this limitation, we complemented IVW with sensitivity analyses (Sections 2.4.4–2.4.6) (Fang et al. 2024).

For forward MR (depression → TBI), we utilized 171 SNPs associated with depression (*P* < 5 × 10⁻⁶, F‐statistic > 10) to maximize instrument strength. In reverse MR (TBI → depression), the relaxed threshold (*P* < 5 × 10⁻⁶) was necessitated by the small TBI sample size, yielding eight SNPs. Both fixed‐effects and multiplicative random‐effects IVW models were implemented. Fixed‐effects IVW assumes homogeneous SNP effects, whereas random‐effects IVW incorporates heterogeneity through a multiplicative variance component. The consistency between these models was critical for validating causal estimates.

#### 2.4.2 Addressing Heterogeneity: Cochran's Q Test

Heterogeneity among SNPs was evaluated using Cochran's *Q* statistic (Qu et al. 2023). A significant *Q* value (*P* < 0.05) indicates inconsistent causal effects across SNPs, potentially arising from pleiotropy or population stratification. In reverse MR, initial heterogeneity (*Q* = 29.399, *P* < 0.001) was resolved after outlier removal via MR‐PRESSO (final *Q* = 2.773, *P* = 0.596). This step ensured that IVW estimates were not biased by SNP‐specific anomalies.

#### 2.4.3 Detecting Pleiotropy: MR‐Egger Intercept and MR‐PRESSO

To evaluate horizontal pleiotropy, we employed two complementary approaches:

MR‐Egger: This method estimates an intercept term representing average pleiotropic bias. A non‐zero intercept (*P* < 0.05) suggests directional pleiotropy. In our analyses, intercepts were non‐significant (forward: *P* = 0.661; reverse: *P* = 0.874), supporting the validity of IVW assumptions (Ouyang and Dai 2023).

MR‐PRESSO: This test identifies outlier SNPs distorting causal estimates using a simulation‐based approach. We applied MR‐PRESSO with default parameters: 3000 iterations for outlier detection and a significance threshold of *P* < 0.05 for the global test. Outliers were removed iteratively until heterogeneity and pleiotropy were minimized, enhancing result robustness (Ouyang and Dai 2023).

#### 2.4.4 Sensitivity Analyses: Mode‐Based and Likelihood‐Based Methods

To address residual pleiotropy and weak instrument bias, we implemented:

Weighted mode: Weighted mode analysis prioritized high‐strength SNPs (F > 10) from the 171 depression‐associated variants to reduce pleiotropic bias. For example, in forward MR analysis, weighted mode assigned higher weights to SNPs with stronger associations (F > 10) to prioritize their contribution to the causal estimate, thereby reducing bias from pleiotropic variants that might independently affect both depression and TBI.

Penalized weighted median: The penalized weighted median method down weights SNPs with extreme causal estimates, thereby reducing bias from invalid instruments. This approach was particularly critical in forward MR analyses to address potential weak instrument bias arising from the relaxed SNP threshold (*P* < 5 × 10⁻⁶).

Maximum likelihood: Models SNP‐exposure and SNP‐outcome associations probabilistically, accounting for measurement error. This method improved precision in reverse MR by accommodating noise in the TBI GWAS summary statistics.

#### 2.4.5 Validation: Leave‐One‐Out Analysis

Stability of causal estimates was confirmed by sequentially excluding individual SNPs and recalculating IVW estimates. No single SNP disproportionately influenced results in either forward or reverse MR, demonstrating robustness against instrument variability (Jin et al. 2024).

#### 2.4.6 Statistical Software and Reporting

All analyses were performed using the TwoSampleMR package (v0.6.5) in R. OR with 95% CI were reported for binary outcomes. Consistency across methods (IVW, weighted mode, penalized weighted median) and sensitivity analyses (heterogeneity, pleiotropy, leave‐one‐out) was required to establish bidirectional causality.

## 3 Results

### 3.1 Effect of Depression on TBI

**FIGURE 2:**
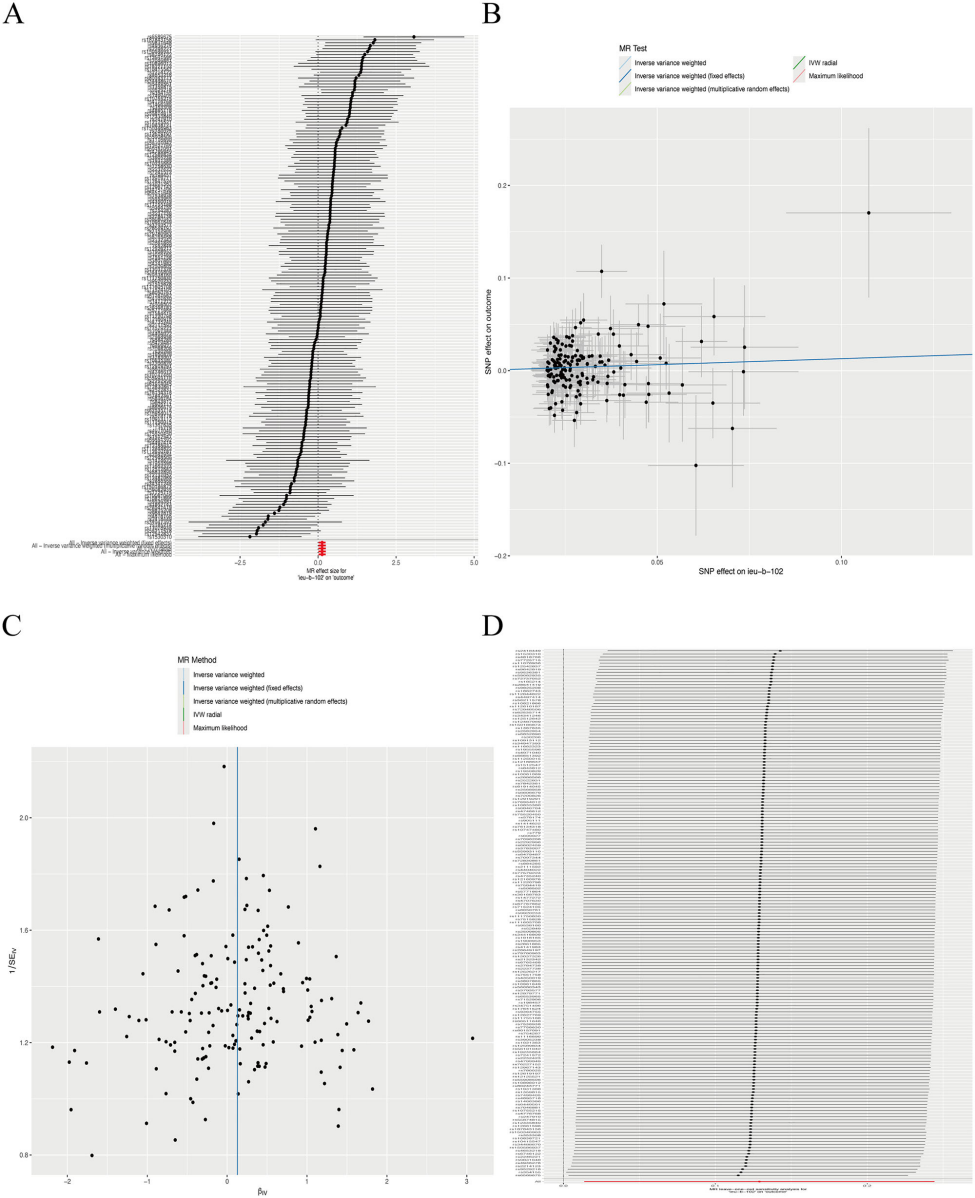
Results of forward MR (depression→TBI) analysis. (A) forest plot displays causal effect estimates (OR with 95% CI) for each SNP and pooled results from multiple MR methods (IVW, fixed‐effects IVW, multiplicative random‐effects IVW, IVW radial, maximum likelihood); (B) scatter plot illustrates SNP effect associations between depression (exposure) and TBI (outcome), with colors denoting different analytical methods; (C) funnel plot tests symmetry of SNP effect estimates, indicating no significant heterogeneity; and (D) leave‐one‐out analysis confirms result stability, with OR fluctuations ≤ 0.01 after excluding individual SNPs.

As presented in Table 1 and Figure 2, our analysis revealed a significant causal effect of depression on the risk of traumatic brain injury (TBI).

**TABLE 1:** MR results for the causal effect of depression (exposure) on TBI (outcome).

Number of instrumental SNPs for depression: 171			
MR method	Odds ratio (OR)	95% Confidence interval(CI)	*P*‐value
**Inverse variance weighted (IVW)**	1.138	1.014–1.277	0.028
**Maximum likelihood**	1.137	1.017–1.274	0.024
**IVW radial**	1.137	1.014–1.277	0.028
**IVW (multiplicative random effects)**	1.137	1.014–1.277	0.028
**IVW (fixed effects)**	1.137	1.019‐1.271	0.022
**Cochran's *Q* (heterogeneity)**	—	—	0.209
**MR‐PRESSO (horizontal pleiotropy)**	—	—	0.218
**MR‐Egger intercept (horizontal pleiotropy)**	—	—	0.661

*Note*: *Leave‐one‐out analysis indicated that after excluding any single SNP, the fluctuation range of OR was ≤ 0.01, *Instrumental SNPs for depression were selected based on genome‐wide significance (*P* < 5×10⁻⁶) and F‐statistic > 10.

**Abbreviations**: CI, confidence interval; IVW, inverse variance weighted; MR, Mendelian randomization; OR, odds ratio; SNPs, single‐nucleotide polymorphisms.

The IVW analysis demonstrated a significant positive association between depression and traumatic brain injury (TBI), identifying depression as a risk factor for TBI (OR = 1.138, 95% CI = 1.014–1.277, *P* = 0.028). This finding was consistently supported by supplementary analytical approaches: maximum likelihood (OR = 1.137, 95% CI = 1.017–1.274, *P* = 0.024), IVW radial (OR = 1.137, 95% CI = 1.014–1.277, P = 0.013), IVW (multiplicative random effects) (OR = 1.137, 95% CI = 1.014–1.277, *P* = 0.028), and IVW (fixed effects) (OR = 1.137, 95% CI = 1.019–1.271,*P* = 0.022). Sensitivity analyses revealed no significant heterogeneity (Cochran's *Q* = 184.662, *P* = 0.209) or horizontal pleiotropy (MR‐PRESSO: *P* = 0.218; MR‐Egger intercept: *P* = 0.661). The robustness of the results was further confirmed by leave‐one‐out analysis, which showed no substantial changes when individual SNPs were sequentially excluded. (Table 1)

These findings collectively demonstrate a credible positive association between depression and TBI, with depression serving as a significant risk factor for TBI. The consistency across methodologies and stability in sensitivity analyses strengthen the validity of these causal inferences.

### 3.2 Effect of TBI on Depression

**FIGURE 3:**
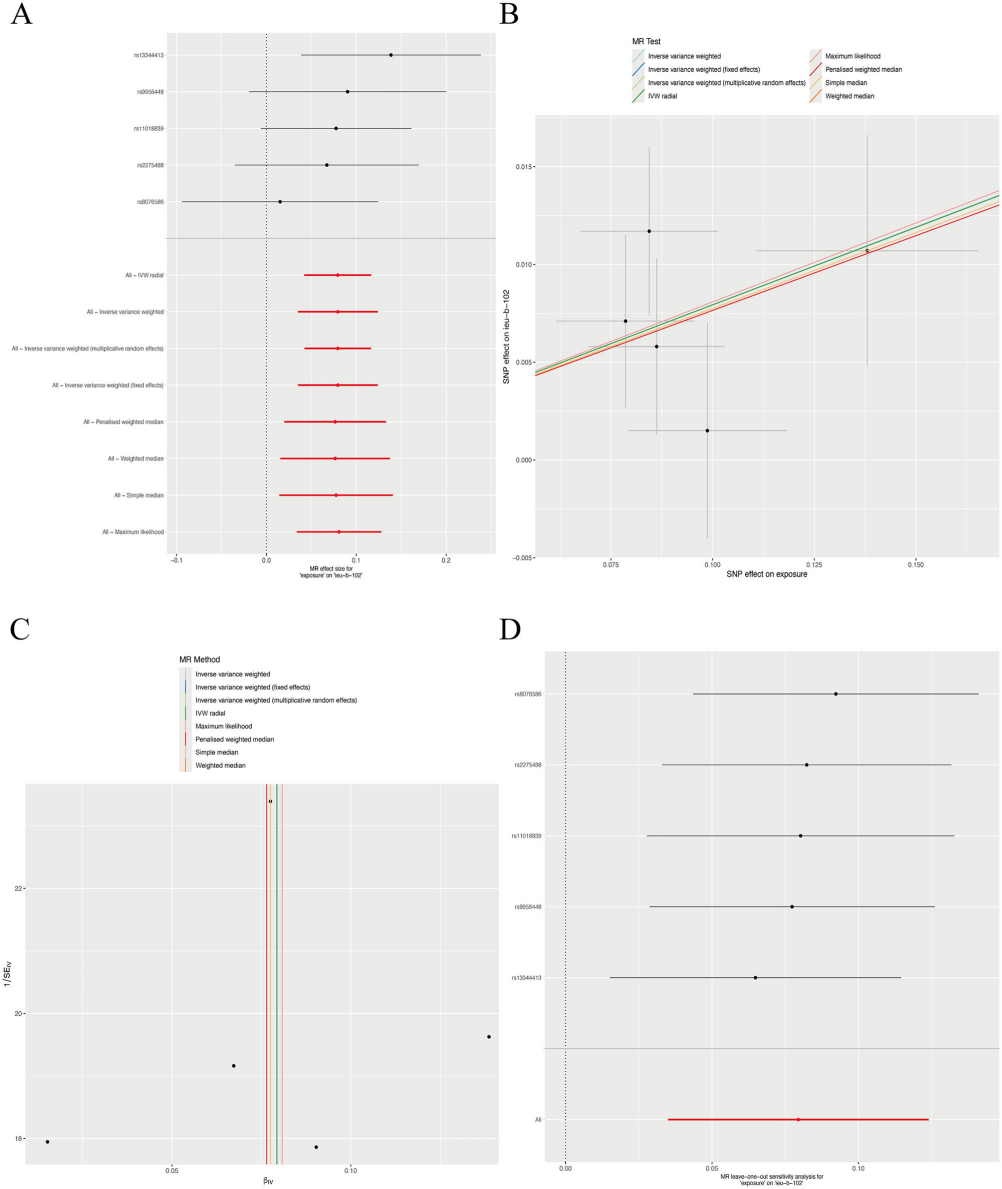
Results of reverse MR (TBI → depression) analysis. (A) forest plot presents causal effect estimates (OR with 95% CI) for each SNP and pooled results from MR methods (fixed‐effects IVW, simple median, weighted median, penalized weighted median, IVW radial); (B) scatter plot demonstrates SNP effect associations between TBI (exposure) and depression (outcome), with colors indicating analytical methods; (C) funnel plot assesses heterogeneity, which was resolved after outlier removal via MR‐PRESSO (final Q = 2.773, P = 0.596); and (D) leave‐one‐out analysis validates result stability, showing OR fluctuations ≤ 0.01 after excluding individual SNPs.

As shown in Table 2 and Figure 3, our analysis revealed a significant causal effect of TBI on the risk of depression.

**TABLE 2:** MR results for the causal effect of TBI (exposure) on depression (outcome).

Number of instrumental SNPs for TBI: 8			
MR method	Odds ratio (OR)	95% Confidence interval(CI)	*P*‐value
IVW (fixed effects)	1.083	1.036–1.131	< 0.001
Simple median	1.081	1.015–1.150	0.014
Maximum likelihood	1.084	1.034–1.136	0.001
Weighted median	1.079	1.016–1.147	0.014
Penalized weighted median	1.079	1.018–1.145	0.011
IVW (multiplicative random effects)	1.083	1.043–1.124	< 0.001
IVW radial	1.083	1.043–1.124	< 0.001
Cochran's *Q* (heterogeneity)	—	—	0.596
MR‐PRESSO (horizontal pleiotropy)	—	—	0.672
MR‐Egger intercept (horizontal pleiotropy)	—	—	0.874

*Note*: *Leave‐one‐out analysis indicated that after excluding any single SNP, the fluctuation range of OR was ≤ 0.01, *Instrumental SNPs for depression were selected based on genome‐wide significance (*P* < 5× 10⁻⁶) and F‐statistic > 10.

**Abbreviations**: CI, confidence interval; IVW, inverse variance weighted; MR, Mendelian randomization; OR, odds ratio; SNPs, single‐nucleotide polymorphisms.

The IVW (fixed effects) analysis revealed a significant positive association between TBI and depression, identifying TBI as a risk factor for depression (OR = 1.083, 95% CI = 1.036–1.131, *P* < 0.001). This finding was consistently supported by supplementary analytical approaches: simple median (OR = 1.081, 95% CI = 1.015–1.150, P = 0.014), maximum likelihood (OR = 1.084, 95% CI = 1.034–1.136, *P* = 0.001), weighted median (OR = 1.079, 95% CI = 1.016–1.147, P = 0.014), penalised weighted median (OR = 1.079, 95% CI = 1.018–1.145, *P* = 0.011), and IVW‐derived frameworks (multiplicative random effects: OR = 1.083, 95% CI = 1.043–1.124, *P* < 0.001; radial IVW: OR = 1.083, 95% CI = 1.043–1.124, P < 0.001). Sensitivity analyses demonstrated initial heterogeneity (*Q*  = 29.399, *P* < 0.001), which was resolved after removing outlier SNPs (*Q* = 2.773, *P* = 0.596). No horizontal pleiotropy was detected (MR‐PRESSO: *P* = 0.672; MR‐Egger intercept: *P* = 0.874), and the stability of causal estimates was confirmed by leave‐one‐out analysis. (Table 2)

These results collectively establish TBI as a significant risk factor for depression, with methodological consistency and robust sensitivity analyses reinforcing the validity of this bidirectional causal relationship.

## 4 Discussion

In this study, we collected genetic variant data closely associated with depression and TBI from GWAS databases, conducting comprehensive MR analyses and sensitivity assessments. The forward MR analysis revealed a positive correlation between depression and TBI, identifying depression as a significant risk factor for TBI. Conversely, the reverse MR analysis demonstrated that TBI also exhibits a positive association with subsequent depression development, suggesting bidirectional causality. These findings align with previous observational studies while effectively mitigating potential biases from unmeasured confounders inherent in observational designs. By employing MR methodology, this research provides robust evidence clarifying the causal relationship between depression and TBI. The results emphasize the clinical necessity for physicians to thoroughly evaluate pre‐injury depressive status during TBI management, while encouraging further mechanistic investigations into the pathophysiological interplay between TBI and depression.

TBI primarily results from direct or indirect violent forces acting on the brain, commonly occurring due to accidents, traffic collisions, falls, or physical assaults (Rauchman et al. 2023). In patients with depression, emotional dysregulation may precipitate self‐injurious or suicidal acts (Mendes et al. 2022; Kalin 2020), potentially representing one mechanism through which depression contributes to TBI occurrence. Studies also indicate that depression increases fall risk (Peterson and Kegler 2020), another possible pathway linking depression to TBI. Furthermore, substance abuse among depressed individuals may elevate TBI risk through hazardous behaviors or falls. The mechanisms underlying TBI‐induced depression are multifactorial. Current evidence suggests associations with neuroanatomical and molecular alterations (including prefrontal gray matter reduction, basal ganglia lesions, and frontal cortex damage), elevated proinflammatory cytokines, and dysfunctions in neurotransmitter systems (cholinergic, glutamatergic, dopaminergic, and serotonergic systems) (McGuire et al. 2019; Fakhoury et al. 2021). Post‐TBI psychosocial adaptations—particularly to severe disability, loss of meaningful social roles, social isolation, chronic pain, and fatigue—constitute significant risk factors for depression development in TBI survivors (McGuire et al. 2019; Howlett et al. 2022; Scholten et al. 2016).

This study reveals that depression remains a critical clinical concern both preceding and following TBI onset, warranting heightened attention from clinicians. Current therapeutic approaches for depression include pharmacological interventions (SSRIs/SNRIs, TCAs, and MAOIs), physical therapies (electroconvulsive therapy, transcranial magnetic stimulation), lifestyle modifications (exercise therapy, substance cessation, regular circadian rhythms and balanced nutrition), psychological interventions (cognitive behavioral therapy, interpersonal therapy, mindfulness‐based cognitive therapy, psychodynamic therapy), and psychosocial support systems (family interventions, group therapy) (Silverberg and Panenka 2019; Huntley et al. 2023; Jorge et al. 2016; Ponsford et al. 2016; Feng et al. 2019; Park and Goodyer 2000). Notably, while existing evidence suggests partial efficacy of these interventions in post‐TBI depression, substantial limitations persist regarding treatment outcomes and complication profiles. A recent umbrella review synthesizing 16 years of evidence concluded that no pharmacological interventions for post‐TBI depression are supported by high‐quality evidence, highlighting the urgent need for novel therapeutic strategies (Hicks et al. 2023). While pharmacological interventions (e.g., SSRIs) are commonly prescribed, recent meta‐analyses highlight limited efficacy and high heterogeneity in treatment responses for post‐TBI depression, underscoring the need for personalized approaches integrating psychotherapy and neuromodulation. The SNPs identified in our study may influence depression pathogenesis through dominant, recessive, or X‐linked inheritance patterns by modulating gene expression or protein function. Investigating causal relationships between these genetic variants and depression through subsequent gene expression alterations and protein functional changes could unveil novel therapeutic targets, potentially informing innovative gene therapies and pharmacological developments for depression management.

In conclusion, our study establishes a bidirectional causal relationship between depression and TBI. Specifically, depression may act as a potential risk factor for TBI development, while TBI could subsequently serve as an etiological contributor to depression onset.

## 5 Limitation

This study has several methodological considerations: (1) genetic instrument assumptions: The relaxed TBI threshold (*P* < 5 × 10⁻⁶) reflected sample size constraints (*n* = 3193; estimated IVs < 10 at *P* < 5 × 10⁻⁸), though sensitivity analyses (MR‐PRESSO, F > 10) validated instrument strength (Burgess et al. 2013); (2) population generalizability: European‐ancestry focus limits extrapolation to populations with distinct gene‐environment landscapes (e.g., high‐trauma incidence regions) (Chen et al. 2020); (3) dynamic confounding: Unmeasured time‐varying factors (e.g., post‐TBI socioeconomic shifts) and developmental windows (e.g., childhood trauma) may persist; (4) bidirectional mechanisms: While suggesting mutual causation, the predominant pathways (e.g., microglia‐mediated neuroinflammation vs. TBI‐induced social isolation) need longitudinal biomarker verification; and (5) methodological boundaries: Residual pleiotropy risks warrant triangulation through multivariable MR (adjusting for neuroimmune markers) and cis‐SNP colocalization.

Collectively, these limitations delineate critical boundaries for applying MR‐derived causality in clinical decision‐making, especially regarding the temporal instability of depression‐TBI interactions observed in real‐world trauma populations.

## Author Contributions

**Shiping Wang**: writing ‐ original draft; conceptualization; methodology; writing ‐ review and editing. **Lei Pan**: writing ‐ review and editing. **Binyang Wang**: software; resources; investigation; validation. **Qianwen Ruan**: formal analysis; resources. **Ying Shi**: formal analysis. **Tong Sun**: formal analysis. **Xu Yang**: formal analysis; visualization. **Lei Zhang**: data curation. **Xiaohua Ke**: formal analysis. **Geng Li**: resources. **Meihua Qiu**: writing ‐ review and editing; data curation. **Chuanxiong Li**: funding acquisition; writing ‐ original draft; writing ‐ review and editing; project administration; supervision.

## Institutional review board statement

Ethical authorization was deemed unnecessary for the purpose of this research.

## Informed consent statement

As the data from the GWASs were publicly available, and all primary investigations were granted specific ethical approval and informed consent.

## Conflicts of Interest

The authors declare no conflicts of interest.

## Peer Review

The peer review history for this article is available at https://publons.com/publon/10.1002/brb3.70669.

## Data Availability

The data that support the findings of this study are available in GWAS at https://gwas.mrcieu.ac.uk. These data were derived from the following resources available in the public domain: ‐ https://gwas.mrcieu.ac.uk., https://gwas.mrcieu.ac.uk.
